# From the Catastrophic Objective Irreproducibility of Cancer Research and Unavoidable Failures of Molecular Targeted Therapies to the Sparkling Hope of Supramolecular Targeted Strategies

**DOI:** 10.3390/ijms24032796

**Published:** 2023-02-01

**Authors:** Irina Alekseenko, Liya Kondratyeva, Igor Chernov, Eugene Sverdlov

**Affiliations:** 1 Shemyakin-Ovchinnikov Institute of Bioorganic Chemistry of the Russian Academy of Sciences, 117997 Moscow, Russia; 2 National Research Center “Kurchatov Institute”, 123182 Moscow, Russia

**Keywords:** cancer, cell–cell interaction, synapses, biomolecular condensate, microenvironment, tumor heterogeneity

## Abstract

The unprecedented non-reproducibility of the results published in the field of cancer research has recently come under the spotlight. In this short review, we try to highlight some general principles in the organization and evolution of cancerous tumors, which objectively lead to their enormous variability and, consequently, the irreproducibility of the results of their investigation. This heterogeneity is also extremely unfavorable for the effective use of molecularly targeted medicine. Against the seemingly comprehensive background of this heterogeneity, we single out two supramolecular characteristics common to all tumors: the clustered nature of tumor interactions with their microenvironment and the formation of biomolecular condensates with tumor-specific distinctive features. We suggest that these features can form the basis of strategies for tumor-specific supramolecular targeted therapies.

## 1. Fundamentally Low Reproducibility in Cancer Research

“One of my biggest frustrations as a scientist that it is so hard to know which exciting results are sturdy enough to build on”, is how Dr. Yusuf A. Hannun, director of the Stony Brook University Cancer Center in New York, reacted in his comment in *Nature* [1] to the recently published results of a large-scale replication project that highlighted just how hard it is to repeat results, even those published in high-impact papers [2].

A group of researchers was involved in an eight-year project to reproduce the findings of more than 50 high-impact papers [3]. It was launched in 2013 by the nonprofit Center for Open Science (COS) in collaboration with the online research marketplace Science Exchange. It was designed to assess reproducibility in preclinical cancer research and attempted to reproduce key results from more than 50 high-impact studies published between 2010 and 2012.

Over the next eight years, the researchers managed to repeat experiments from a little under half of those studies and found that the results they obtained were typically far less clear-cut than the ones reported in the original papers—an assessment that has drawn criticism from some of those papers’ authors. The team was often not able to obtain enough information about the methods used from either the papers or their authors and had to abandon attempts at replication altogether.

Finally, in December 2021, a series of papers appeared [4,5,6] that described reproducibility issues in high-impact cancer papers [3] and identified significant challenges associated with repeating other scientists’ work, renewing calls for increased transparency and data-sharing in the biomedical community.

The problem was reviewed in detail by us earlier [7] and even before by Begley and Ioannidis in a paper entitled “Reproducibility in science: improving the standard for basic and preclinical research” [8]. These authors underlined an important problem: “The estimates for irreproducibility based on these empirical observations range from 75 to 90%...”. The variability of biological systems means that we should not expect an obligatory reproduction of the results to the smallest detail. Many people are amazed, not by the fact that scientists cannot exactly reproduce experiments, but that, in many cases, the main conclusion is not supported even when the same scientists conduct repeated, but blind, experiments when information about the test and control is masked (kept) from the participants. Empirical assessments of preclinical studies have revealed many other problems, including the studies that were not repeated, incorrect control usage, reagent quality not being tested, and incorrect statistical tests not being used. In addition, researchers often choose the best result for publication instead of a summation of the whole set of data. Commonly, the result of such a practice is that not only are some experiments not reproduced, but the main conclusion of the paper is not confirmed.

A recent review added more information in this regard: In a survey of 1500 researchers, about 70% of those who attempted to reproduce someone else’s experiment failed; additionally, and even more worrisome, more than 50% failed to reproduce their own experiments. The problem is not limited to cancer. For the most recent consideration of this problem with non-cancerous diseases, see [9].

It should be noted that the problem of reproducibility in cancer research is not new. Countless steps have been taken to solve it: better reporting, better career incentives, separating exploratory work from confirmatory work, and developing infrastructure for large, collaborative confirmatory experiments [1]. Barriers to reproducing preclinical results also included unhelpful author communication [10].

However, all the reasons listed above are subjective and can indeed be largely minimized by the correct organization of experiments and rules for publishing results. The situation is much worse with the objective, intrinsic variability of biological objects in general, which is especially high in cancerous tumors.

Due to the application of fast methods for decoding the whole genomes of organisms, as well as the genomes of individual cancer tumors, it became clear that cancer development is associated with hundreds of human genes. The mechanisms underlying the association of genomic changes and cancer phenotype development were understood only to a negligible extent. The unpleasant truth is that the majority of associations between the genotype as a genome structure and the phenotype as a combination of external traits are extremely complex. Genes are mutually dependent, they upregulate or downregulate the effects of each other, and every phenotype manifestation involves many genes and non-genetic processes that are called epigenetic. Identical genotypes in different conditions produce different phenotypes, whereas different genotypes can behave in a similar way. This is true not only for cancer but also for other diseases [11].

This multidimensional complexity results in extreme genetic, epigenetic, transcriptional, and proteomic cancer heterogeneity. One gene may have many phenotypes, and at the same time, one phenotype may be formed by many genes [12]. As such, Mullard [10] was absolutely right when he noted an extremely important problem: “You can never do experiments exactly the same”.

In this short review, we will look at these objective causes of variability in more detail, trying to highlight some general principles in the organization and evolution of cancerous tumors. We think that, in the case of cancer, the modern medical “sacred cow”, personalized medicine, in view of the extreme heterogeneity of cancers, encounters serious difficulties in its application and formulae; this is in contrast to possible generalized principles of cancer medicine. Such approaches seem especially relevant against the backdrop of a rapidly growing population, an equally rapidly deteriorating ecology, which increases the incidence of cancer, and the very likely inaccessibility of personalized medicine to developing countries and individuals. Under such conditions, big medicine must concentrate on universally applicable treatments.

## 2. Heterogeneity within Cancer Cells and in Their Interactions with Microenvironment

One of us considered this problem earlier [13,14]. Since then, quite a number of reports have confirmed the points of view expressed there. For the most recent reviews, see, for example, [15,16,17]. In brief, there are more than 100 distinct types of cancer in specific organs [18]. A tumor is a growing and evolving system [19] and undergoes multiple changes to become cancerous, to resist antitumor agents, and to induce inter- and intratumoral cellular heterogeneity that makes them unique for every patient [20]. The dozens of “driver” genetic mutations and up to 12 different pathways can be involved in the development of a single cancer type [21].

In addition, cancer cells actively interact with neighboring normal cells by modifying them, forming tumor microenvironments, and by evolving together (see below).

Despite enormous complexity, all cancers have common essential alterations in cell physiology (“hallmarks”) that are collectively necessary (but not sufficient, see below) to order malignant growth [18,22,23]. Each hallmark can be acquired in different ways in different tumors [23]. The permanently growing data demonstrates more and more complex systems of genes, epigenetic changes, proteins, and various complexes involved in tumor initiation and progression. Every cancer cell is a unique entity that possibly emerges only once in the history of mankind [14]. Tumors possibly start from a stem cell converting into a cancer stem cell (for details, see [14] and references therein). The latter divides into two daughter cells, and these three cells are different from each other according to a number of genetic changes, due to stochastic mutations and recombinations. In normal tissues, the mutation rate varies from less than 1 × 10^−8^ per base pair per cell division to 10^−10^–10^−11^ in the stem cells, which are supposed to give rise to cancers [14]. During development, cancer cells may exhibit a mutator phenotype [24] (for a recent review, see [25]) that has increased rates of mutagenesis. The mean frequency of mutations in cancer cells was estimated as 2.1 × 10^−6^ [26] (not everyone agrees with the mutator hypothesis, see [14] for discussion).

Considering the number 2.1 × 10^−6^, we will find that if the tumor size is 10^9^ (it is a detectable size—1 g [27] (30 cycles of division occur in a hypothetical scenario in which there is no cell death)), then each cell in such a tumor will have 189,000 mutations. These figures agree rather well with experimental estimates; some cancer genomes carry 100,000 point mutations, whereas others have fewer than 1000 [28]. With such a number of randomly distributed mutations, the distribution of these mutations along the genome is unique in each particular cell of 10^9^ different cancer cells. All of the cells are different from all others in this particular tumor, and as so in all other tumors in the world [14,15,16,29,30].

The tumor heterogeneity is further increased due to epigenetic [31], proteomic [32], and metabolomic [33] heterogeneity detected, in particular, by single-cell omics means [34,35,36].

A huge amount of information concerning tumor heterogeneity obtained by means of single cell omics technology has been published recently. Researchers use methods to test individual cells, from characterizing their gene expression to documenting their epigenetic state, transcription factor activity, and cell–cell communication [37]. Numerous problems exist with wet-lab techniques and adequate analytical methods. The reproducibility of the results is also questionable [38,39]. As the primary goal of our review is to show the extreme variability of cancers at both the intercellular and intracellular levels in order to explain the failures of molecular targeted approaches in cancer therapy, a discussion of the reliability and reproducibility issues of single cell omics extends beyond the scope of this review. The reader can read about this extremely intensively developing field in the latest comprehensive reviews and comments, as detailed in [38,39,40,41,42]. Intracancerous heterogeneity determines the heterogeneity of molecules and complexes exposed to the surface of cancer cells (surfaceome—all of the surface proteins of a cell or organism [31,43]; The Cancer Surfaceome Atlas integrates genomic, functional, and drug response data and substances secreted by cancer cells [44]). For the understanding, identification, and therapeutic treatment of cancer, some of the most important components of the surfaceome are a plethora of adhesion molecules, which, in turn, have sometimes been united under the term adhesome [45].

Cell adhesion molecules (CAMs) play central roles in much of the connection and communication between cells. Cell-adhesion-related communication is essential for the correct development in a variety of organs and tissues and also plays a substantial role in cell recognition processes in adult organisms [46]. Surface adhesion proteins are connected to a network of cytoskeleton proteins, implementing signal transduction.

The secretome of an organism [47] represents the proteins and other products released by all types of cells/tissues of the organism. A cell secretome represents all the substances secreted by the cell [48]. Secretory Proteins (SPs) are crucial for correct cell proliferation, metabolism, immune functions, and communication. Many SPs serve as important biomarkers for diverse cancers, and some of them could be used as therapeutic targets.

All of these variable factors form complex systems with differences between cancer cells in various aspects of gene expression, phenotypic markers, growth dynamics, and, importantly, unpredictable emergent properties that make the tumor extremely resistant to therapeutic interventions. In addition, cancer evolution leads to metastasis and circulating tumor cell clusters (see below), which are formed due to the cancer–tumor microenvironment (TME) interactions. On the other hand, the same cancer–TME interactions (hereinafter defined as intratumoral) necessary for its evolution can serve as its Achilles heel, at which killing arrows can be directed. The network of these interactions could be defined as cancer connectome in contrast to intracellular interactions defined as the interactome [49].

Below we try to consider various aspects of the complexity of tumor systems, which make each tumor a unique formation and lead to objective reasons for the poor reproducibility of studies conducted in this area. We focus on systems of cancer cells–TME connectomes and put forward a hypothesis that the destruction of these interactions can be a universal strategy for tumor therapy [50].

## 3. Tumor Micro Environment (TME) and Its Immunological Components (TIME) Heterogeneity

Tumor heterogeneity determines the heterogeneity of the tumor microenvironment (TME), including the tumor immune microenvironment (TIME), of cancer cells.

In our previous reviews [51,52,53], we tried to outline the problems of the tumor microenvironment. Here, we briefly repeat some points discussed there, add important new information that has appeared since these publications appeared, and focus mainly on the immune component of tumor stroma.

The definition given by the American National Cancer Institute to the TME is as follows: “The normal cells, molecules, and blood vessels that surround and feed a tumor cell. A tumor can change its microenvironment, and the microenvironment can affect how a tumor grows and spreads” [54]. Cancer cells are the primary architects of the tumor microenvironment [55]. During tumor evolution, cancer cells use the tumor–stroma crosstalk to reorganize the microenvironment for maximum tumor robustness.

The TME consists of a complex mixture of various cells and extracellular material. TME cells, including fibroblasts, cancer-associated fibroblasts (CAFs), myofibroblasts, mesenchymal stem cells, adipocytes, and endothelial cells, have a mesenchymal origin and cells of hematopoietic origin, such as lymphoid cells (T, B, and NK cells) and myeloid cells (macrophages, neutrophils, and myeloid-derived suppressor cells), that form TIME (see above). The non-cellular component is represented by the extracellular matrix. All of these entities form an interacting and evolving system with multiple emergent properties. They also recruit normal cells and form an ecological tumor niche—a very important player in both the development of the primary tumor and its metastasis [56,57,58,59,60,61,62].

The interactions of cancer and stromal cells include (i) direct binary contacts between ligands and receptors exposed on the surface of cancer and stromal cells, and (ii) paracrine communication between cancer cells and various TME cells [53,63,64]. Some authors use the term “symbiotic” for tumor–stroma interactions, such as [52] and the references therein. The symbiosis of cancer and stromal cells is based on a complementary exchange of paracrine factors that leads to changes in the TME characteristics, the most important result of which is the transformation of normal fibroblasts into cancer-associated fibroblasts (CAFs) [65,66,67,68,69,70,71,72,73]).

Paracrine signals can be transmitted by diffusion over distances of tens of cell diameters [64], forming a gradient of signals that exclude the “yes” or “no” binary responses of the cells but, depending on the distance from the source, induce different responses. Clearly, the signal efficiency will be higher for closely located cells, where it occurs in synapse-like structures (see below). The tumor–stroma crosstalk eventually leads to the increased robustness of the tumor. A hypothesis was put forward [51] that such interactions include the formation of synapses and synapse-like structures with the interacting cells positioned at a distance of 10–30 nm [74]. Such a tight intercellular space could enhance the paracrine cross-communication.

TIME has been shown to be significantly involved in tumor development and metastasis and is highly heterogeneous [75,76,77,78,79,80].

The important role of immune system cells in the progression of cancer and, in particular, metastases has been repeatedly noted (see, for example, review [58] and the references therein). It was reported, in particular, that the immune system can augment secondary tumor growth. In a mice model, the recruitment of monocytes/macrophages and neutrophils in TIME advanced tumor cell survival, colonization, and pre-metastatic niche establishment was observed. Neutrophils, in their turn, can enhance metastasis by grouping Circulating Tumor Cells (CTCs) in circulation. They can also promote metastatic growth by remodeling the host extracellular matrix.

In their comprehensive review [81], the authors provide information concerning the pro- and antitumoral role of various immune cells present in the TIME. In particular, the authors note that CTC and white blood cells can be clustered during circulation (see below), or even earlier, and that the important participants of this process were innate immune cells [82]. Many authors also mention macrophages as one of the most abundant cells in TIME, the presence of which correlates with worse survival in most cancers, and they participate in many stages of cancer evolution [83]. Other cell–cell interactions in metastatic clusters were also reported [84].

We will return to the problem of immune cell participation in tumor progression, but to conclude this paragraph, we want to emphasize once again that inter-patient heterogeneity in immune composition and immune cell function is also clearly manifested. It represents a major challenge for cancer immunotherapy [85].

## 4. General Principles of Intercellular Interactions

Clustering is a prominent feature of receptors and ligands in the plasma membrane (PM). It plays an important role in signaling [51].

Extracellular protein–protein interactions for soluble ligands are rather strong, with the equilibrium constant of dissociation (Kd) in the nanomolar to the picomolar range. Such high-affinity binding ensures signal initiation under conditions when the concentration of interacting molecules in the solution is low. On the contrary, the affinities are extremely low for membrane-bound individual receptor–ligand–protein interactions, with the Kd bound within the micromolar to the millimolar range [86]. This effect is due to the very low half-life of the membrane-embedded protein contacts that often have milliseconds in a monomeric state [87]. The strength of intercellular contacts, in this case, is achieved through the clustering of adhesion molecules that involves hundreds of ligand–receptor pairs. This increases the avidity of the intercellular contact to a level sufficient to switch on a signaling cascade [88].

**Figure 1:**
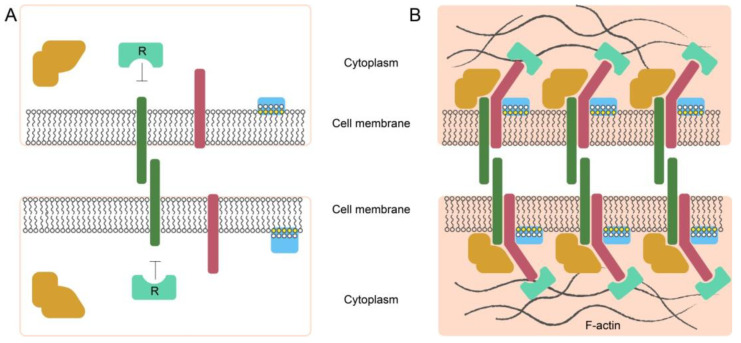
Illustration of individual molecules freely diffusing on the membrane surface (**A**), and a cluster of the intercellular adhesive complexes (**B**). Adhesion molecules (deep green) initiate binding, which also may involve other transmembrane proteins (pink) and cytoplasmic proteins that can bind to the cytosolic part of the transmembrane proteins (orange). It also involves lipid groups present on the inner surface of the plasma membrane (yellow) and proteins with lipid-binding domains (light blue). Clustering may lead to the displacement of negative regulators associated with the cytosolic part of the adhesion molecules (R). Actin microfilaments stabilize macromolecular clusters through actin-binding proteins (cyan) (modified from [91]).

A relatively well-studied example of cell–cell interactions through surface ligand–receptor pairs is the clusterization of cadherins during the formation of the cadherin-mediated intercellular contacts [89]. The emergent intercellular adhesion is initiated by the binding of cadherin ectodomains to the cell surfaces. Due to diffusion, the formed cadherin trans-dimers gather into small clusters at the sites of cell adhesion. With the participation of intracellular transformations of the cytoskeleton formed by the inner parts of the cadherins, the clusters are stabilized and expanded. As a result, cell adhesion is enhanced strongly. Small nanoclusters usually slowly diffuse or can be fixed through the actin cytoskeleton. Upon the binding of a ligand, the already existing small nanocluster can include accessory monomers (Figure 1). The activation of the nanoclusters through binding ligands leads to an enlargement of nanoclusters, making them functional. Nanoclusterization is a general organizing principle for many membrane receptors. Nanoclusters often coexist with randomly distributed non-clustered components. This coexistence may play a functional role or a regulatory role. Nanoclusters may function as complexes assembled in advance and are capable of fast activation when binding a ligand [90].

### 4.1. Synapses as a Way of Communications of Tumor and Its TIME

Synapses are adhesive spaces between two neighboring cells in multicellular organisms. They serve cell–cell communication as well as information processing and storage.

The synapse concept appeared more than 100 years ago for neuronal cell–cell communication [92,93] and recently was adapted to other cell–cell communication mechanisms. Currently, the concept of the synapse is used to describe various intercellular communications [94], among them are specialized adhesive contacts of various types of cell–cell interactions, such as neurons, immune cells, epithelial cells, and even pathogens and host cells [95,96,97,98,99,100,101,102,103,104].

The history of many failures demonstrates the very low effectiveness of cancer treatment targeted at indecipherable intracellular interactomes, and the development of efficient cancer therapies should focus on a new paradigm. A classic example of a new paradigm can be demonstrated by immune checkpoint therapy, which focuses on the interactions between cancer and stromal cells as therapeutic targets. As we tried to substantiate earlier [51,52], only direct interactions (for example, between ligands and their cognate receptors) form relatively simple binary contacts that are necessary for successful therapeutic action. Such binary contacts universally provide synapse-like structures (interfaces) where the interacting cells are located at a distance of 10–30 nm (see below). Within these interfaces, molecules initiating and strengthening the interaction are organized, and a very confined intercellular space facilitates the concentration of secreted cytokines, enhancing paracrine cross-communication. These features of synapses represent a new target for efficient cancer drug discovery [51,52]. A telling example of the success of such a concept is tumor immune checkpoint therapy.

### 4.2. Immunological Synapse as a Classical Example of Cell–Cell Functionally Efficient Interactions

**Figure 2:**
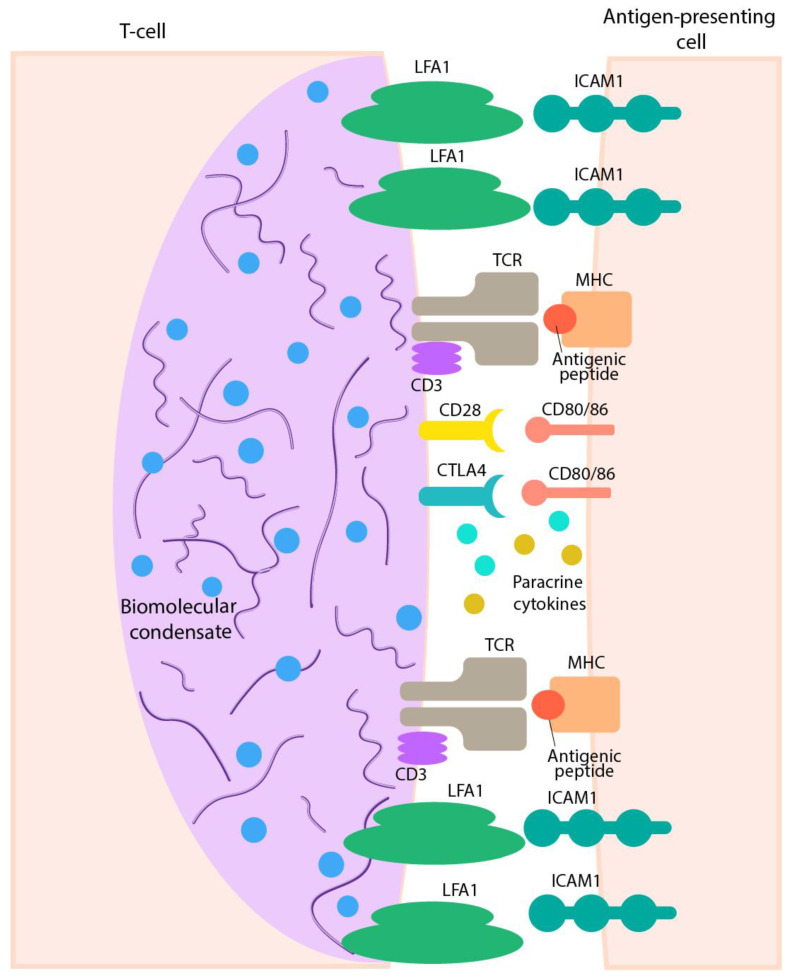
Simplified representation of the organization of the immunological synapse (IS) demonstrating a T-cell receptor (TCR)/CD3 complex-MHC bound peptide interactions within the synapse cleft. Clusterization of other receptors (CD28, CTLA4 with their ligands (CD80) and adhesion molecules LFA-1 and ICAM-1, on the surface of both cells, are responsible for the formation and stabilizing ISs and for the initiation of signal pathways generated by the TCRs [95,103,109]. All transmembrane contacts are clustered and have been symbolized by their pairs in the figure. Synapse formation also leads to the appearance of a biomolecular condensate (see below).

The most important example of clusterization to make the cell–cell contact strong enough is the enabling of the immunological synapse to form a stable interface between the immune cells organized by the adaptive or innate immunoreceptors in concert with adhesion molecules [100,105]. One of the most important functions of the immunological synapse is the integration of innate and adaptive signals to decide if the initiation of an immune response or an effector program is appropriate following specific antigen recognition [100]. A synapse formed by a T-cell receptor (TCR) is illustrated in Figure 2. Micro-clusters interacting with ligands and receptors/co-receptors, as well as adhesive molecules, are formed and create a stable 10–30 nm cleft allowing for effective exchange with cytokines and other substrates. Synaptic clefts are densely occupied with different proteins, such as adhesion proteins, receptors, and transporters [74].

Stable synapses are important in the regulation of immune response decisions and efficient effector function [100].

Cell–cell adhesion is mediated by structurally diverse classes of cell-surface glycoproteins, which form homophilic or heterophilic interactions across the intercellular space [94,105]. Surface-positioned adhesion proteins have internal parts that bind to a cytoplasmic network of scaffolding proteins, regulators of the actin cytoskeleton, and signal transduction pathways that control the structural and functional organization of synapses.

As indicated above, an essential feature of the ISs is the formation of surface-receptor and ligand clusters, which mediate intercellular contacts. Some authors even suggest the formation of synapse-like structures for all cases of membrane signalization. For example, as indicated in [106], “this in a way predicts a ‘synapse’ like entity for all membrane signaling events. Here, there is no difference between a ligand/receptor pair induced higher-order lipid domain or one produced by a membrane curvature or any other biophysical means. The central purpose is to bring together enough sorted lipids and their associated protein receptors, and signaling ensues”.

A receptor cluster in the T-cell synapses initiates the recruitment of hundreds of molecules to the membrane, interacts with the actin cytoskeleton, and plays a significant role in signal transmission. The formation of signal clusters leads to functional results that are difficult to predict from individual components [107]. These complex system interactions lead to the appearance of numerous emergent properties [108].

Clusterization provides stability for signaling by enhancing ligand–receptor functional local concentration and reducing the possible effect of the protein-degrading enzymes on the interaction result. A fundamental property of the synapse is the proximity of the interacting cells. Such proximity was reported in an X-ray structural analysis of a CD200R and CD200 protein complex. CD200 (earlier known as OX2) is a widespread cellular surface protein that interacts with the receptor CD200R, expressed in the myeloid cells and some lymphoid cells. The authors calculated a distance of ~12 nm between the interacting cells, which corresponds to the spatial parameters of an immunological synapse. Since CD200 is also expressed in the non-lymphoid cells, synapse-like interactions may be widely used [110,111]. Various techniques provide a width of the immunological synaptic cleft ranging from 10 to 30 nm [74].

The NKG2D receptor abundantly present on all NK cells is important for immunological synapse formation between NK and tumor cells. NKG2 allows NK cells to recognize virus-infected cells and tumor cells [112,113] and to form the cytotoxic synapse, antibody-stimulated proliferation and adhesion of NK cells to target cells [114].

### 4.3. Other Systems also Use Synapse-like Contacts for Cell–Cell Communication

In the last ten years, the synapse concept was adapted to embrace other cell–cell communication phenomena [92]. Intercellular interactions underlie multicellularity. The key components that mediate these interactions are now known, in particular: the cadherin superfamily, nectins, CAMs, connexins, Notch/Delta, lectins, and eph/Ephrins [115]. Below we will briefly consider some of them, forming synapse-like structures.

#### 4.3.1. Ephrin Type-A Receptor 2 (EphA2)/EphrinA1 System

The transmission of intercellular adhesion signals in other cellular systems is similar to the processes in T-cell immunological synapses. The ephrin type A receptor 2 (EphA2)/EphrinA1 system, which regulates cell adhesion, motility, and angiogenesis, is one recent example. Surface-attached cell ligand ephrins convey signals through receptors that are members of the Eph family of tyrosine kinases. Dimerization in receptor tyrosine kinase signaling is well known [116]; however, to trigger Eph signaling, higher-order oligomerization is necessary [117]. The binding of EphA2 to EphrinA1 eventually leads to the formation of clusters on the cell membrane [107].

Ephrins and Ephs signal complexes may change cluster size and composition. It has been reported [118] that the receptor is initially activated by the formation of 6-mer to 8-mer oligomers. The interactions between these oligomers can further form larger clusters that inhibit signaling [119]. This system uses fundamentally the same organizational principles as the immunological synapse: intercellular contacts are achieved due to the presence of receptor clusters on one of the interacting cells and ligand clusters on the other. These clusters are associated with the remodeling of the intracellular cytoskeletons. This allows the polarization of the cell secretory mechanism—another feature of synapse-directed secretion [103,106,110,120].

#### 4.3.2. Phagocytic Synapse-like Behavior

Macrophages (MPs) in TME or tumor-associated macrophages (TAMs) cells are critical elements in regulating tumor development in multiple cancers [83,92,121,122,123]. MPs are one of the most abundant immune cells in the TME, and their presence may indicate an unfavorable development of the disease. MPs participate throughout the whole tumor evolution and stimulate angiogenesis, invasion, and the intravasation of the metastatic cells at the primary site and participate in the preparation of a metastatic site for the arrival of metastatic cells and the promotion of their subsequent growth [83]. An important function of macrophages is the elimination of injured or apoptotic cells [124].

MPs can *engulf* tumor cells and present tumor-specific antigens for recognition by the cells of the adaptive immune response. In addition, macrophages also rapidly recognize and engulf apoptotic cells (efferocytosis) in the tumor microenvironment, which inhibits inflammatory responses and facilitates the immune escape of tumor cells [121].

The term “phagocytic synapse,” analogous to the immunologic synapse, has been used to determine the space between phagocyte receptors and their target [122,125]. The generation of phagocytic and immunological synapses is initiated by different immune receptors and executed by different cell types. However, it was demonstrated that very similar molecular and cellular events control both processes. Phagocytic and immunological synapses use specific patterns of receptors, signaling molecules, and clustering of phagocytic receptors to induce signal transduction pathways using particle-associated ligands. Phagocytic receptor signaling switches on the reorganization of the actin cytoskeleton similarly to the one induced by the TCR. The list of similarities can be continued, as detailed in [122].

Phagocytic synapses, as well as other synapses, explain the high potential of eukaryotic cells for the integration of multitudes of various signals into desirable physiological effects.

Recent studies argue that MPs and TAMs can enter the cell cycle and self-renew [126]. The plasticity of MPs and their ability to self-renew significantly influences tumor progression and resistance to therapy. TME factors also influence macrophage metabolism and can have a significant impact on TAMs proliferation [127].

Innate immune cells (MPs, neutrophils, NK cells, etc.) detect and eliminate non-self invaders. Unlike adaptive immune cells, innate immune cells use a limited number of receptors, recognizing certain structural pattern characteristics of an invader or damaged cells; these are the so-called pattern recognition receptors (PRRs). Receptor clustering has been discovered in the activation of the innate immune system during host–pathogen interactions. It has been reported that innate immune receptors also assemble into nano- or micro-sized domains on the surfaces of cells, forming a multi-component system for the detection of harmful events and organizing a proper immune response [128,129].

#### 4.3.3. Viral (Virological) Synapses

A viral (virological) synapse is a cellular junction that has the characteristic features of a synapse. Viruses such as the herpes simplex virus (HSV), human immunodeficiency virus (HIV), and human T-lymphotropic virus (HTLV) form these junctions between the infected (“donor”) and uninfected (“target”) cells and, in such a way, achieve direct cell–cell viral transmission; thus, they escape destruction by the immune system [130,131,132].

#### 4.3.4. Gap Junctions

Gap junctions (GJ)—a fundamental structure of normal epithelial cell function are clusters of intercellular channels found in immunological synapses.

GJs form a trans-cellular channel formed by connexins [133] for the efficient direct exchange of ions, metabolites, and second messengers. Heterogeneous GJs are important to normal growth and differentiation [134,135,136,137]. GJs serve as an important communication between tumor cells and stromal cells. Increased GJs coupling blocks metastatic potential in some cancer–animal models, such as breast cancer and melanoma [135]. There exists plentiful evidence suggesting that connexins, in particular connexin-43 (Cx43) gap junctions, regulate signaling events in different types of IS [138]. In particular, GJs accumulate at the immunological synapse [139] and contribute to T-cell activation at the cytotoxic immunological synapse [140]. It was noted that the role of GJs in tumor evolution is ambiguous; independent of GJ composition and cancerous factors, tumoral GJ may support tumor progression or suppression [137,141,142]. In addition to gap junctions, cells in the epithelial state also use tight (TJ) and anchoring junctions (AJ) for cell–cell interactions, which can be further subdivided into adherens junctions, desmosomes, etc. [143]. Additionally, recent research indicates that some molecules in cell–cell junction structures have little effect on primary tumor initiation and growth, but they are instead critical for the formation of distant metastases [143].

## 5. Metastasis

Metastasis is responsible for more than 90% of cancer-related deaths globally [144]. Malignant tumor cells in TME are maintained and/or antagonized by their existing immune cells [145,146,147,148,149,150,151]. These interactions also initiate metastasis, which involves the detachment of tumor cells from their primary site and intravasation into the circulation. They then survive there, migrate, and extravasate into the secondary organ to survive and grow at the new site [144,152]. CTCs (circulating tumor cells) exist as single cells or cell clusters (groups of aggregated CTCs, also known as the circulating tumor microemboli [153]). The process also includes epithelial–mesenchymal transition (EMT) in single cells and a hybrid EMT in collective migratory cells [150,154,155]. EMT is a key step in the metastasis of tumor cells that lose polarity and acquire the migration ability necessary to metastasize [156]. E-cadherin is expressed mainly in epithelial cells. It functions within the AJs of the epithelial junctional complex and helps the cells to form a polarized cell layer performing barrier and transport functions. This is essential to the stabilization of cell–cell adhesion. E-cadherin loss leads to the increased invasiveness and metastatic potential of cells [142]. Some data indicates that TAMs induce the EMT program to enhance CTC migration, invasion, and CTC-mediated metastasis [157].

Only a small proportion of the single CTCs (0.2% reported by Tripathi et al. [158]) can survive and result in metastatic transformation [159]. Efficient metastasis (>90% [158]) has been attributed to the CTC clusters. According to the estimates, per day, 3.2 × 10^6^ tumor cells per gram of primary tumor can be detached. More than half of them die [153]. Only one cell per 10^6^–10^7^ leukocytes [153,160] remains. Clusters in circulation reportedly have 20- to 50-fold greater metastatic potential, but a shorter half-life (6–10 min for clusters vs. 25–30 min for single cells), higher proliferation rate, and distinct molecular features compared to single CTCs. Their presence in the peripheral circulation is associated with unfavorable clinical outcomes in cancer patients [161]. Perhaps some role in the efficiency of metastasis is played by the supposed acquisition by circulating cells of the properties of cancer stem cells [162]. The CTC can evade immune surveillance [161,163,164,165,166,167,168,169].

CTC clusters that incorporate tumor or non–tumor cells, such as neutrophils, platelets, macrophages, MDSCs, natural killer (NK) cells [170], and CAFs [171], may be homotypic [172,173] or heterotypic. In particular, tumor-associated macrophages (TAMs), major components of the tumor microenvironment, are frequently associated with tumor metastasis in human cancers [157]. The interactions between immune cells and CAFs with cancer cells possibly produce signaling activities, promoting cancer invasion and metastasis [51,57,173,174,175,176].

The non–tumor cells can combine with cancer cells through multiple cell-adhesion molecules and tight junction proteins both in homotypic and heterotypic CTC clusters [51,153,166,167,169,177,178,179,180].

The interactions between neoplastic and immune cells (possibly by means of synapse formation) regulate different stages of the metastatic process. Immune cells contribute to invasion by secreting a large number of inflammatory factors that promote epithelial-to-mesenchymal transition and the remodeling of the stroma. Above we have already mentioned that paracrine-secreted signals have a rather small probability of reaching the target cell due to diffusion [64]. The formation of a synapse would allow these signals to be focused through a narrow synaptic cleft and make the signal efficiency for closely located cells much higher. Cancer cells then intravasate to the circulatory system assisted by macrophages and use several pathways to avoid recognition by cytotoxic lymphocytes and phagocytes [181,182].

Recently, the presence of hybrid TAM and glioblastoma (GBM) cells that possessed higher invasiveness was detected [147]. Additionally, macrophage-like cells containing phagocytosed tumor material (CAMLs) and circulating hybrid cells (CHCs) that probably result from cell fusion between cancer and immune cells were found in circulation [146]. Both can play a role in the metastatic cascade and are presented in higher numbers as compared to CTCs in the peripheral blood. It was shown that macrophages displayed the most robust cell fusion capacity [146]. Tumor-immune hybrid cells harbor immune and neoplastic cell attributes. The role of such cellular griffons is not yet clear.

In their comprehensive review, the authors of [164] (also, see references therein) discuss several important points. The formed groups of cells can arrange themselves in such a way that, at the front-most end of the cluster, “leader” cells directing migration connected to several “follower” cells appear. Leaders probably determine the direction of the cluster migration. “Tip” cells are connected by cell–cell junctions to “stalk” cells leading to the multicellular partners of endothelial cells. Despite their importance, there is no consensus on how leader cells arise or their essential characteristics [149]. The number of cells in each group and their composition vary greatly; however, this leader–follower combination has repeatedly been observed. Leader and follower cells can communicate directly through cell–cell junctions or through secreted molecules. Leader cells generate a migration path, coordinate cluster cells to facilitate collective movement, and enhance the survival and metastatic potential of the tumor [151]. TAM, CAF, basal epithelial leader cancer stem cells, mesenchymal leader cancer stem cells, hybrid, and EM leader cancer stem cells were considered as possible leader cells [151].

CTC clusters at least partially retained epithelial characteristics, in particular, the high expression of cell–cell junction proteins and gap junction formation [183]. The authors [164] note that cell–cell adhesion helps cells avoid death by activating integrin signaling without ECM. In tumors, closely positioned cell-cluster signals can be directly transmitted to their nearest neighbors (synapse?), and adjacent tumor cells can also form gap junctions, allowing the diffusion of signaling molecules directly between the cytosols of the contacting cells. It was reported [184] that the development of epithelial cancers is mostly proceeded by the collective invasion of cell groups with coordinated cell–cell junctions and multicellular cytoskeletal activity. The gap junction’s ability to permit direct cytosol–cytosol flow makes them, and, possibly, synapses, promising targets to disrupt tumor cell–cell communication [185].

**Figure 3:**
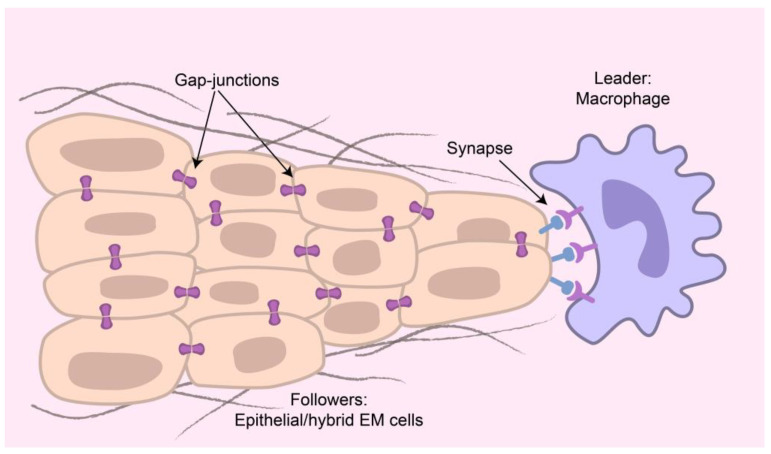
Hypothetical picture of a circulating tumor cell (CTC) cluster with a leader macrophage cell connected by synaptic interactions, with epithelia/hybrid mesenchymal–epithelial followers interacting with each other by means of gap junctions.

An open question is whether synapse formation may play some role in the collective movement of CTC clusters, as is shown in Figure 3. It would be reasonable to assume that, by performing “professional” functions, macrophages (or other immune cells) could contact tumor cells that send danger signals and, acting professionally, form a phagocytic synapse with them. The strength of this connection would be provided by the clustering of receptors and adhesion molecules. Additionally, the properties of such a ligament could be determined by the highly probable formation of an MC (see next paragraph) in the synapse [186,187]. The lifetime of such a connection would be long enough to survive traveling through the vasculature. For estimation: cytotoxic lymphocytes (CTLs) form somewhat transient synapses, lasting only a few minutes, as the target cells are killed. On the other hand, Th lymphocytes make stable, lengthy synapses (>20–30 min up to several hours) that are necessary for both directional and continuous secretion of stimulatory cytokines [96,160,188].

To conclude this section, it could be argued that intercellular cooperativity represents the main driving force behind the invincibility of metastasis. Therefore, anti-cancer and anti-metastatic drugs should be aimed at breaking these sinister alliances.

Another conclusion in the context, indicated at the beginning of the article, is the objective irreproducibility of cancer; it is clear that both the tumor microenvironment and the composition and properties of metastases are unique for each patient. However, the interactions used by the tumor are based on common principles. Additionally, these common principles should be universal anti-cancer targets.

Similar thoughts have already been expressed [52,189,190]. The two major concerns in such an approach are: which interactions are the most common and most vulnerable, and how do we destroy them in the safest possible way for a patient. We will consider these issues below.

## 6. Biomolecular Condensates (Membrane Less Compartments)

It is evident now that multiple biochemical processes in cells take place in so-called membraneless compartments (MCs), otherwise called biological biocondensates or membraneless organelles [141,180,187,191,192,193,194,195,196,197,198,199].

MCs have been described in bacteria and in mammalian cells, both in the cytoplasm and in the nucleus. These compartments are not enclosed in a membrane; they can rapidly form and dissolve as well as easily change their properties. Firstly, such organelles, nucleolus, and Cajal bodies were noticed as early as in the 19th and early 20th centuries. In the cytoplasm, MCs, stress granules, P bodies, and germ granules were observed considerably later. The reactions occurring in the MCs are essential for diverse areas of cell functioning, including transcription, stress response, synaptic activity, and many more. There are convincing arguments (though not proof of) that the condensation of molecules into MCs may play important roles in various human diseases, such as cancer and viral infections, numerous neurodegenerative disorders, and other diseases [200]. The basic rules determining the formation, behavior, and physiological functions of MCs are still in the process of detection and evaluation. For recent information on databases related to this problem, the reader will find it in [195].

**Figure 4:**
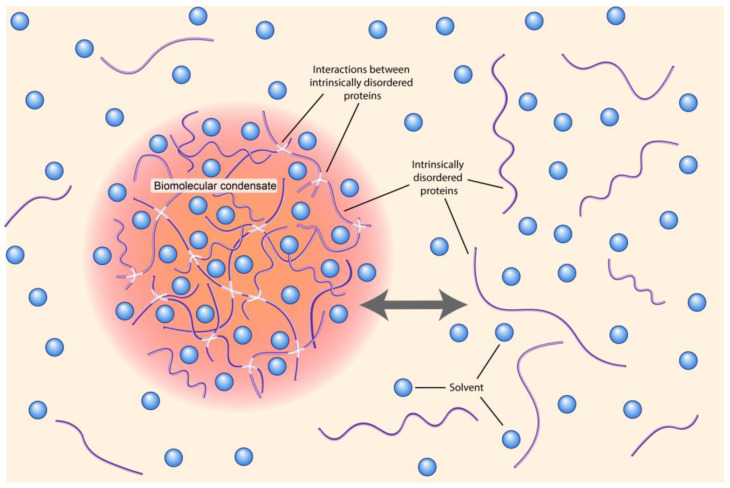
Liquid−liquid phase separation of intrinsically disordered proteins (IDS) into biomolecular condensates (modified from [196]).

Due to a process of liquid–liquid phase separation (LLPS), sequestering specific proteins and nucleic acids into MCs takes place. As a result, molecules in solution form a condensate phase with high molecule concentration and a surrounding phase with a low concentration of molecules (Figure 4) [196]. The last decade was full of reports concerning this problem, and many excellent reviews have summarized the results and hypotheses relating to their possible roles in development, cancer and neurologic diseases [193].

MC formation depends on a solute concentration. As soon as it reaches a critical threshold, the system experiences a phase division into two phases: concentrated and dilute. MCs reversibly form in response to various cellular signals, in particular, changes in local concentration and epigenetic and post-translational modifications, and are stabilized by numerous weak biomolecular interactions [201].

Modest perturbations can result in fundamental shifts in a phase-separating system [191,193,202,203]. Biological phase separation is governed, in part, by noncovalent weak, transient multivalent and dynamic interactions among biopolymers, in particular, proteins and nucleic acid polymers. Whereas classical biochemical complexes have a defined stoichiometry, condensates are non-stoichiometric assemblies with a great number of molecules with weak multivalent interactions, which are self-organized via clustering [202]. MCs allow for the organization of several billion proteins, nucleic acids, and other molecules of the cell into separate compartments of the cell with specific functions [204]. A separate MC usually contains tens to hundreds of macromolecules, though only a small part of them appears to participate in the formation, structural integrity, and function of the MC [195].

MCs are dynamic structures that can condense or dissolve rapidly. Such properties allow MC to respond efficiently to changing conditions [205,206], in particular, by adopting the regulation of gene expression and signaling [207].

Compartmentalization can significantly enhance the rate of biochemical reactions. Many cellular processes have recently been shown to occur in biomolecular condensates [204]. MCs gather various factors involved in shared processes [208]. For example, transcription involves a plethora of various biomolecules that should be arranged in a functionally active transcriptional complex. Compartmentalization allows this goal to be reached, and assembling multiple transcription factors together with the RNA-polymerase at the promoter of a gene ensures a high level of transcription [204,209,210,211,212,213]. Transcriptional condensates form at enhancers and promoters containing multiple transcription-factor (TF) binding sites through selective TF binding [165,204,209]. It is important that cytoplasmic condensates can form around the plasma membrane signaling apparatus [180,186,204,214,215,216].

MCs form and dissolve quickly enough. Due to this property, the cells have the possibility to collect and release biomolecules for use in different places after they are exempt from use at a previous site [193,208,217]. Probably, one of the central roles of phase separation is to quickly respond to fluctuations in the surrounding milieu.

It should also be noted that MC and membrane-bound organelles interact with each other and are dynamically regulated by the cellular signaling network [218], a complete understanding of which is still (or forever?) very far from understanding.

MC composition depends on its specific location in the cell; nuclear condensates can contain DNA- or RNA-binding proteins, whereas cytoplasmic condensates can form at sites on the plasma membrane [186,204,210,217,219]).

Individual MCs are densely populated with certain protein and RNA molecules that are enriched for specific sets of biomolecules and depleted in others. In addition, proteins that contain repetitive domains, and that are involved in the formation of transmembrane signaling complexes, for example, clusters of T-cell receptors, are known to be inclined toward MC formation [187]. The transmembrane signaling proteins of receptors can be core components essential for MC formation [180]. MCs, as a rule, contain from ten to several hundred various proteins and/or RNA molecules. Some components are constitutive, and others are recruited transiently, for example, in response to stimuli. The contents of condensates are chemically distinct microenvironments [191,202,220]. As a rule, molecules inside and outside the condensate exchange across the boundary [220].

The principles regulating MC formation were discussed recently [191,214,221]. The authors [221] introduced the concept of the “the molecular connectivity” of an MC. The number of weak protein–protein interactions per unit of volume, determining the stability of the MC, was positively correlated with connectivity. The molecules whose connectivity govern the formation and existence of an MC (so-called “scaffolds” [191]) are supplemented by molecules called “clients” [191], recruited by means of interactions with scaffolds.

Various mechanisms for the regulation of MCs have been suggested. For example, in [222], two mechanisms have been proposed to explain MC formation: 1) folded domain (secondary structures such as α-helices or β-strands) exploitation and/or 2) the utilization of intrinsically disordered regions (IDRs; unfolded regions that do not contain secondary structures, as shown in Figure 4 [180,186,214,223]. In the first mechanism, proteins containing tandem folded domains can be bound through protein–protein interactions. An illustrative example of such a mechanism is that it provides the formation of nephrin clusters using multivalent interactions between phosphotyrosines and Src homology 2 (SH2) domains and between proline-rich motifs and SH3 domains [186]. This was the first example of successful reconstitution of phase-separated condensates in vitro. A similar mechanism involving crosslinking operates when DNA or RNA molecules are used instead of the protein components.

Proteins that form scaffolds of MC typically have numerous intrinsically disordered regions (IDR) [196,208,224,225]. IDRs play a key role in the formation and properties of MC [193,196], though mechanisms of the phase transition driving are still unknown. IDRs are often depleted of hydrophobic residues and enriched with polar and charged residues. They do not have a certain conformation in solution but rapidly exchange between different conformations, thus, forming dynamic complexes [193]. The proteins with IDRs are rather usual in the human proteome (~35% of all amino acids are predicted to be disordered), and their sequence conservation in evolution is rather low. However, IDR functions are often preserved across large evolutionary distances in the regulation of transcription, translation, and signaling [193]. Client proteins cannot form MC; they bind to available sites in the scaffolds, thus finalizing MC assembly [191,208].

Distinct proteins and nucleic acids involved in common functions occur in each individual condensate. This variability probably indicates the distinct chemical specificities of these compartments. This suggestion is supported by the observation that different small-molecule drugs concentrate in different condensates. The term ‘chemical grammar’ has been proposed recently [226] to describe the rules determining the chemical features of small molecules that cause attraction or repulsion due to the chemical content of an individual condensate. If such rules exist, knowledge of them could help in the design of drugs that target the compartment with therapeutic purposes.

## 7. P.S. Overcautious Fear

As interest in LLPS as a mechanism for organizing macromolecules in cells has increased, as have criticisms of the quality of evidence for LLPS in vivo [227,228,229]. Some researchers have expressed doubt that “the evidence for in vivo LLPS is often phenomenological and inadequate to discriminate between phase separation and other possible mechanisms”. Moreover, they fear that the causal relationship between the formation of condensates and their functional consequences is not well established [227].

## 8. Instead of a Conclusion: Persistent Therapeutic Supramolecular Targets in Cancer’s Fickle Microcosm

Numerous failures in clinical trials concerning targeted agents gave rise to a legitimate question: is the paradigm of molecular targeting correct? The theoretical considerations and findings discussed above suggest that it is barely possible to develop an efficient therapy targeted to specific genes [14,230,231,232,233,234]. Tumorigenesis involves practically all cells of the stromal environment of cancer [235]. Disrupting these detrimental connections is a challenging, but still achievable and promising, task. To defeat cancer, we must admit the insurmountable complexity of intracellular interactomes and try to disrupt the system as a whole by destroying the interactions of its parts, connectome.

Direct cell–cell interactions produce targets simple enough to expect real therapeutic effects. A bright example of the success of such a concept is a paradigm of tumor check-point immunotherapy ([52,236,237] and references therein).

However, this approach confronts other sides of tumor heterogeneity—quite a number of patients do not respond to this therapy—and the inhibition of immune checkpoints causes numerous side effects called immune-related adverse events (IRAEs) [238,239,240]; furthermore, the problem of resistance that occurs during treatment is also a matter of serious concern. Deborah Madden [241] asks if cancer immunotherapy entails a change in paradigm? She writes that though immunotherapy had become the best achievement in the field of cancer in 2016–2017, it spawned more questions than answers. Further investigations into tumor–TME interactions are necessary for the identification of new immune checkpoints or other key interactions that allow for more reliable therapeutic strategies [84,242].

The survival of cancerous cells at all stages critically depends on the interactions between each other and their microenvironment or with neighbors in the circulating tumor cells during metastatic migration (see above). The means of cell–cell communications are highly diverse meaning that even the gap distance between the plasma membranes of two cells varies independent of communication type. For example, tight junctions between epithelial cells have a gap size of ~2 nm, whereas the synaptic cleft in the case of an immunological synapse is 10–30 [74]. However, cell–cell contacts share a common set of components [141], and many of them are composed of large clusters (large-scale molecular assemblies) of proteins at the cell membranes (see above and [243]), which are especially essential in cancer. Each cluster recruits hundreds or even thousands of signaling adhesion proteins to the membrane outside of the cell and numerous molecules of the actin cytoskeleton inside of it (see above). These clusters have one common property: their components are located close to each other, and with different quantitative compositions, they have similar qualitative features. It should be noted that an extremely important feature of clusters is that their components interact cooperatively [141].

**Figure 5:**
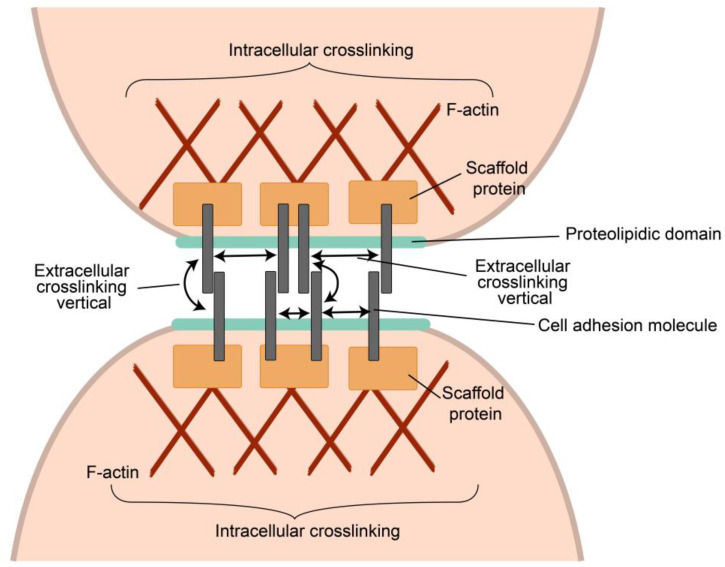
Schematic illustration of application of bi-valent cross-linkers or ligands for modification of clusters connecting the interacting cells.

A detailed analysis of the interactions taking place in CTC clusters demonstrates the dynamic alterations in adhesion molecules and ligand–receptor pairs during the circulation of CTM [84]. In particular, during an invasion, epithelial–mesenchymal transition (EMT) occurs and epithelial cell–cell adhesion proteins, for example, E-cadherin and EpCAM, are usually downregulated due to which tumor cells lose epithelial properties and gain mesenchymal characteristics, such as migratory abilities. Additionally, intermediate hybrid EMT cells appear simultaneously, expressing epithelial and mesenchymal markers. Such hybrids retain some degree of epithelial cohesion, while acquiring enhanced migratory and invasive potential, allowing invasion despite intact intercellular cohesion. Taken together, these results reveal a highly complicated picture [84]. In our recent review [51], we suggest the possible formation of synapse-like structures that emerge during the interaction of cancer cells and cells of TIME, such as macrophages, neutrophils, NK-cells, and the cancer-associated fibroblasts, which could play the role of leaders in the circulating clusters, as shown in Figure 5. Such a combination would use clusters of receptor–ligand and adhesion molecules, which is quite natural for immune cells. The existence of clusters opens a new dimension in cancer treatment. The proximity of adhesion molecules in clusters in itself opens up new possibilities for therapeutic agents directed at nearby receptor–ligand pairs in the clusters. For example, the application of bivalent ligands composed of two functional pharmacophores linked by a spacer. Bivalent ligands are thought to preferably target and stabilize pre-formed/constitutive heteromers [244]. This is considered in pharmacology as one of the promising strategies for the treatment of homo- or heterodimeric receptors (see, for example, [244,245,246,247,248]). Such a therapy may be a new method for tumor destruction. The above pertains to cancerous tumors and their metastasis, and this may supplement the immune checkpoint therapy, which is also targeted at disrupting the synapses between the cancer cells and the cells of the immune system.

Quite reasonable attention has been paid by many authors to the potential therapeutic use of the aberrant behavior of condensates in cancer.

Correlations between changes in phase separation and carcinogenesis only recently attracted the attention of researchers, and, now, growing evidence is accumulating that cancer-associated mutation or post-translational modification may influence the aberrant location, composition, and physical properties of the MC [187,193,194,208,249,250,251,252,253,254]. Given the enrichment for IDRs in proteins related to signaling and gene regulatory functions, which are important for malignancy, authors [193] found that of the 53 of the 287 genes with annotated cancer involvement participate in LLPS. In addition, compared to other diseases, proteins in MC are more enriched in cancer driver genes; for example, they are involved in the maintenance of genome stability, abnormal proliferation signaling, and the regulation of gene expression. In addition to possible abnormalities in phase separation caused by mutations to single genes, cancer gene fusions that have a strong influence on MC formation were also enriched in IDRs. The authors discuss many other correlations in favor of the involvement of MC in cancer evolution. However, in conclusion, they provide an extremely sober evaluation of the data available. As an assessment of the validity of the conclusions drawn on the basis of these data: the role of the mutations’ influence on MC formation in cancer needs to be systematically evaluated, and systematic efforts are needed to understand the role of cancer gene fusions in phase separation and its role in cancer initiation and progression. The extent to which protein folding and stability promote phase separation activity is largely unknown. All these problems need to be systematically examined [193].

There are numerous problems to be solved on how to thoroughly control phase separation. The specific mechanism of the dynamic behavior and impact of MC on the origin and evolution of tumors is far from being understood [251]. Recently, many profound studies have appeared in connection with this problem [255]. This shows the attention and interest that it causes.

One of the most evident objects attracting the attention of researchers [256] is the transcriptional deregulation characteristic for cancerous transformation. Such deregulation could be caused by the reprogramming of gene-control machinery, RNA polymerase II and its cofactors, and, especially, super-enhancers that are responsible for high expression levels. The chromatin state also plays an important role in gene regulation and expression.

The disruption of pathological condensates may provide new opportunities to treat diseases. Small molecules and RNA therapeutics may display unexpected pharmacology in condensates [192,255].

Phase separation seems to open up several new approaches for potential therapeutic intervention in oncology [193]. One of them may use the differential distribution of a small molecular therapeutic between the dilute and dense phases. It was reported that some MC units might be destroyed by 1,6-hexanediol [193]. Recently drugs such as cisplatin were found to be accumulated within condensates, influencing their pharmacodynamics [257]. Some small molecules were identified to be capable of influencing the phase separation properties of p53 mutants [258].

Mitrea et al. [192] discuss the attractive opportunities to develop therapeutic agents for various diseases by means of targeting MC if it turns out that similar condensates are formed and dysregulated in different diseases. In this case, it will be possible to use a single therapeutic agent for treating a larger spectrum of diseases while, classically, researchers are trying to find an individual single target remedy for every disease.

In conclusion, we outlined two supramolecular characteristics common to all tumors: the cluster nature of tumor interactions with their microenvironment, and the formation of biomolecular condensates with tumor-specific distinctive features. We suggest that these features can form the basis of strategies for tumor-specific therapies.

However, it is understandable that numerous challenges need to be overcome before realistic approaches are developed.

## Data Availability

Not applicable.
